# Cloning, Molecular Characterization and Expression Patterns of *DMRTC2* Implicated in Germ Cell Development of Male Tibetan Sheep

**DOI:** 10.3390/ijms21072448

**Published:** 2020-04-01

**Authors:** Taotao Li, Hongyu Zhang, Xia Wang, De′en Yin, Nana Chen, Lingyun Kang, Xingxu Zhao, Youji Ma

**Affiliations:** 1 College of Animal Science and Technology, Gansu Agricultural University, Lanzhou 730070, China; ttli2018@163.com (T.L.); 18894312250@163.com (H.Z.); wangxiaandzisu@163.com (X.W.); gsauyde@163.com (D.Y.); cnn937820369@163.com (N.C.); 18821622269@163.com (L.K.); 2 Sheep Breeding Biotechnology Engineering Laboratory of Gansu Province, Minqin 733300, China; 3 College of Veterinary Medicine, Gansu Agricultural University, Lanzhou 730070, China; zhaoxx@gsau.edu.cn

**Keywords:** Tibetan sheep, *DMRTC2*, cloning, testis, spermatogenesis

## Abstract

The double sex and mab-3-related transcription factors like family C2 (*DMRTC2*) gene is indispensable for mammalian testicular function and spermatogenesis. Despite its importance, what expression and roles of *DMRTC2* possesses and how it regulates the testicular development and spermatogenesis in sheep, especially in Tibetan sheep, remains largely unknown. In this study, *DMRTC2* cDNA from testes of Tibetan sheep was firstly cloned by the RT-PCR method, and its molecular characterization was identified. Subsequently, the expression and localization patterns of *DMRTC2* were evaluated by quantitative real-time PCR (qPCR), Western blot, and immunofluorescence. The cloning and sequence analysis showed that the Tibetan sheep *DMRTC2* cDNA fragment contained 1113 bp open reading frame (ORF) capable of encoding 370 amino acids, and displayed high identities with some other mammals, which shared an identical DM domain sequence of 47 amino acids ranged from residues 38 to 84. qPCR and Western blot results showed that *DMRTC2* was expressed in testes throughout the development stages while not in epididymides (caput, corpus, and cauda), with higher mRNA and protein abundance in Tibetan sheep testes of one- and three-year-old (post-puberty) compared with that of three-month-old (pre-puberty). Immunofluorescence results revealed that immune staining for DMRTC2 protein was observed in spermatids and spermatogonia from post-puberty Tibetan sheep testes, and gonocytes from pre-puberty Tibetan sheep testes. Together, these results demonstrated, for the first time, in sheep, that *DMRTC2*, as a highly conserved gene in mammals, is essential for sheep spermatogenesis by regulating the proliferation or differentiation of gonocytes and development of spermatids in ram testes at different stages of maturity.

## 1. Introduction

Spermatogenesis occurring in the seminiferous tubule boundaries of mammalian testis is a sophisticated, multistep, and continuous biological event where spermatogonial stem cells undergo mitosis, meiosis, and cell differentiation to generate mature spermatozoa that is needed for the continuation of species [1]. Meiosis is one of the most critical processes during spermatogenesis [2,3], which is required for perpetuation of species and generation of new variation. This process is finely regulated by numerous genes, which express at the transcriptional and translational levels [4,5].

Double sex and mab-3-related transcription factors (DMRT) like family C2 (*DMRTC2*, also termed as *DMRT7*), a member of the DMRT family genes, is involved in the regulation of mammalian sex differentiation [6] and the process of spermatogenesis, particularly meiosis [7,8,9]. Hou et al. [10] reported that NF-Y, a transcript regulator, could activate the transcription of *DMRTC2* in murine testis by binding to tandem CCAAT boxes located in its proximal promoter. In mice, previous studies indicated that targeted deletion of *DMRTC2* gene for males give rise to abnormal spermatogenesis such as meiotic arrest, while females exhibit normal fecundity [9,11,12].

As of now, *DMRTC2* has been reported that it plays crucial roles in spermatogenesis of mammals, such as humans [13,14], cattle [8], and mice [7,9,11]. To the best of our knowledge, however, in sheep, and, especially in Tibetan sheep, a Chinese indigenous breed that is the most common and widespread domestic animals dwelling at the Qinghai–Tibet Plateau, with an elevation of between 3000 and 5000 m [15], so far there are hardly any reports on molecular characteristics, expression, and regulatory roles of the *DMRTC2* gene. This work was therefore performed (*i*) to obtain the full-length coding sequence (CDS) of Tibetan sheep *DMRTC2* gene and analyze its molecular characteristics and (*ii*) to investigate its expression and regulation during sheep testicular development and spermatogenesis. This is of great significance to further elucidate the molecular mechanisms of *DMRTC2* in regulation of spermatogenesis in sheep and other mammals.

## 2. Results

### 2.1. Cloning and Sequence Analysis of Tibetan Sheep DMRTC2 CDS

**Figure 1:**
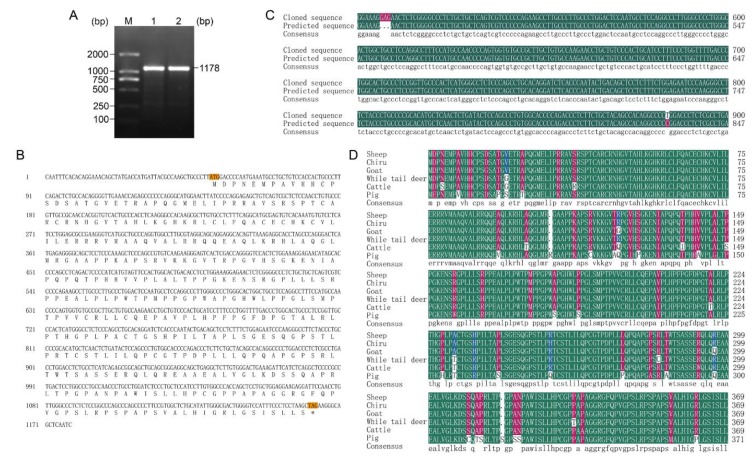
Full-length coding sequence (CDS) cloning and sequence analysis of Tibetan sheep *DMRTC2* gene. (**A**) Results for PCR amplification of *DMRTC2* cDNA. M, DL2000 marker; 1-2, *DMRTC2* RT-PCR product. (**B**) The nucleotide and deduced amino acid sequences for the cloned *DMRTC2* CDS region. (**C**) Comparisons of sheep *DMRTC2* CDS region between the predicted and cloned sequences. (**D**) Alignment of the deduced amino acid sequences of Tibetan sheep DMRTC2 with that of chiru (accession no: XP_005971442.1), cattle (accession no: AAI09621.1), goat (accession no: XP_005692632.1), white tail deer (accession no: XP_020726841.1), and pig (accession no: XP_020950121.1).

A specific target fragment of 1178 bp was obtained by the RT-PCR method using Tibetan sheep testes cDNA as templates (Figure 1A). Sequence analysis showed that the cloned cDNA sequence included a 1113 bp open reading frame (ORF) translatable to 370 amino acid, with an ATG (M) start codon and a TAG stop codon (Figure 1B). The resulting full-length CDS sequence of Tibetan sheep *DMRTC2* has been deposited in GenBank (accession no. MT040732). The CDS sequence alignment showed that the cloned Tibetan sheep *DMRTC2* sequence had three base insertions (GAG) at nucleotide position 457 and one base substitution (C→T) at nucleotide position 831, compared with the predicted sheep *DMRTC2* sequence from the National Centre for Biotechnology Information (NCBI) database (GenBank no. XM_027978391.1; Figure 1C). The amino acid sequence alignment revealed that Tibetan sheep DMRTC2 amino acid sequence displayed 99.73% sequence similarity with chiru, 98.92% with goat, 97.84% with while tail deer, 96.49% with cattle, and 90.32% with pig DMRTC2 (Figure 1D).

### 2.2. Molecular Characteristics of Tibetan Sheep DMRTC2

**Figure 2:**
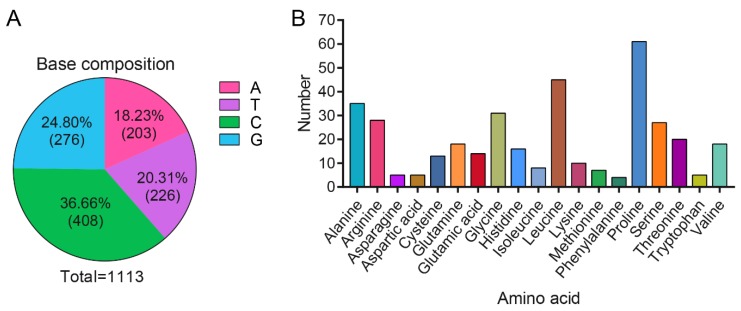
Sequence composition of Tibetan sheep DMRTC2 CDS region at the nucleotide and amino acid levels. (**A**) Base composition. (**B**) Amino acid composition.

At the nucleotide level, *DMRTC2* cDNA sequence was composed of 18.23% base A (203), 20.31% base T (226), 36.66% base C (408), and 24.80% base G (276; Figure 2A). The amino acid composition revealed that proline is the majority amino acid (16.49%), followed by leucine (12.16%), alanine (9.46%), glycine (8.38%), arginine (7.57%), and serine (7.30%; Figure 2B). The isoelectric point (pI), molecular formula and molecular weight for the protein produced by Tibetan sheep DMRTC2 were 9.2, C_1730_H_2796_N_524_O_479_S_20_, and 39.24 kDa, respectively. Tibetan sheep DMRTC2 protein had neither predicted transmembrane region (Figure S1) nor signal peptide sequence (Figure S2). NetPhos 3.1 server analysis based a threshold set (greater than 0.5) revealed that there were 34 potential phosphorylation sites, including 22 serine phosphorylation sites and 12 threonine phosphorylation sites (Figure S3).

### 2.3. The Spatial Structures of Tibetan Sheep DMRTC2 and Evolutionary Relationships Between Sheep and Other Known Mammals

**Figure 3:**
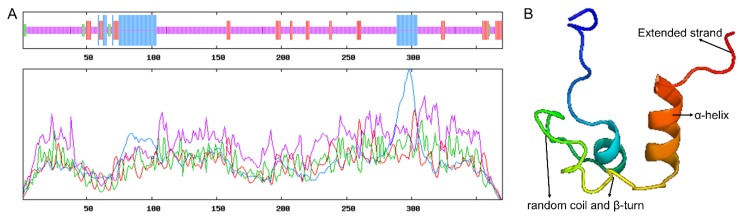
The secondary and tertiary structures of Tibetan sheep DMRTC2 protein. (**A**) Secondary molecular structure. Different lines with different colors denote different secondary structures: blue, alpha helix; red, extended strand; green, beta turn; purple, random coil. (**B**) Tertiary molecular structure.

**Figure 4:**
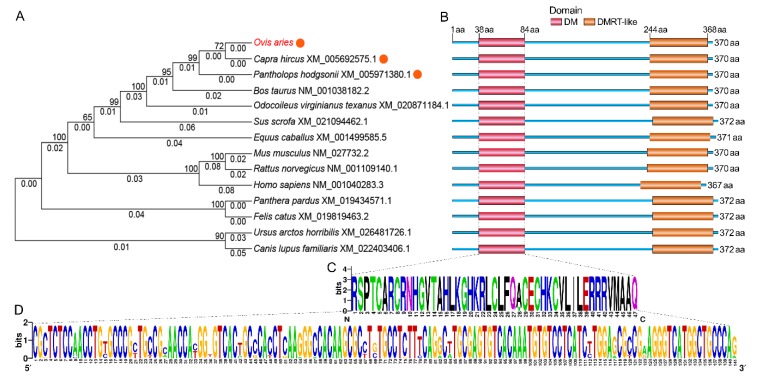
Phylogenetic tree and domain analysis of DMRTC2. (**A**) Neighbor-joining phylogenetic tree based on nucleotide sequences of *DMRTC2* gene among different mammals. The bootstrap values (greater than 50) and branch lengths (less than 1) were showed above and below each branch, respectively. The closest homology with ovine *DMRTC2* is indicated by orange circle. (**B**) DMRTC2 protein domain prediction among different mammals. Weblogo tool was used to show the amino acid (**C**) and nucleotide (**D**) composition of the DM domain.

Secondary structure predictions for DMRTC2 protein showed that it was a protein with mixed secondary structures, including 73.24% random coil, 13.53% α-helix, 11.35% extended strand, and 1.89% β-turn (Figure 3A). The tertiary molecular structure analysis of the coded protein indicated that it was similar with secondary structure, consisting of α-helix, β-turn, random coil, and extended strand (Figure 3B). The phylogenetic tree was constructed based on nucleotide sequences of *DMRTC2* from Tibetan sheep and other known mammals. As seen in Figure 4A, in the evolution, Tibetan sheep *DMRTC2* sequence was the most closely related to goat, followed by chiru and cattle. The conserved domain analysis for DMRTC2 protein indicated that Tibetan sheep DMRTC2 protein contained a DM domain of 47 amino acids long at the amino acid residues 38–84 region close to the N terminus of the protein, as well as a DMRT-like domain of 125 amino acids long at the amino acid residues 244–368 region (Figure 4B). The nucleotide and amino acid sequence analysis corresponding to the DM domain suggested that this domain structure was very well conserved among mammals (Figure 4C,D).

### 2.4. Expression Patterns of DMRTC2 at the Transcript and Protein Levels

**Figure 5:**
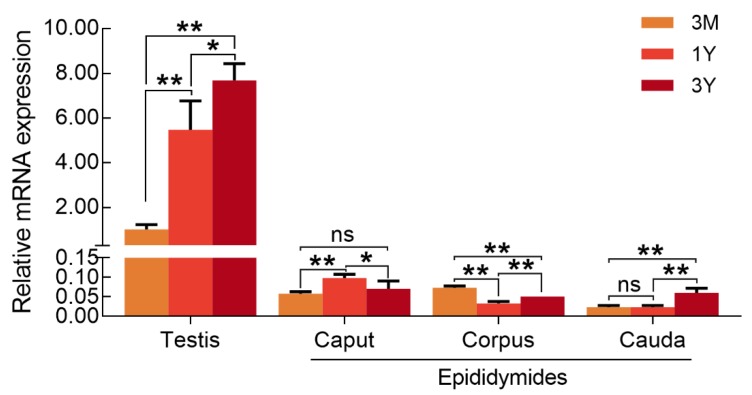
Temporal expression patterns of *DMRTC2* mRNA in developmental testes and epididymides. Data show means ± SD from eight independent experiments. **: *p* < 0.01, *: *p* < 0.05, and ns (no significance): *p* > 0.05. 3 M: three months old, 1 Y: one year old, and 3 Y: three years old.

**Figure 6:**
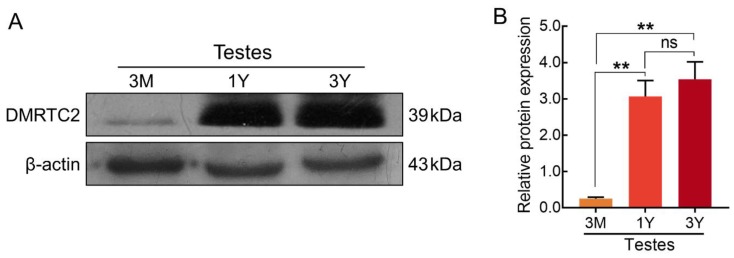
Temporal expression patterns of DMRTC2 protein during testicular development. (**A**) Western blot analysis for DMRTC2 protein. (**B**) Relative expression level of DMRTC2 protein. Data show means ± SD from eight independent experiments. **: *p* < 0.01, *: *p* < 0.05, and ns: nonstatistical significance. 3 M: three months old, 1 Y: one year old, and 3 Y: three years old.

qPCR results showed that *DMRTC2* mRNA expressed in Tibetan sheep testes but not in caput, corpus, and cauda epididymides. Specifically, its expression abundance in testicular tissues progressively increased with age, with the highest level in the 3Y group (Figure 5). At the protein level, DMRTC2 expression in developmental testes demonstrated a consistent trend as the mRNA level (Figure 6). A slight DMRTC2 protein expression was detectable in the 3 M testes, whereas significant DMRTC2 protein was found in the 1 Y and 3 Y testes (Figure 6). The full blot images are shown in Figure S4.

### 2.5. Localization of DMRTC2 Protein in Developmental Tibetan Sheep Testes

**Figure 7:**
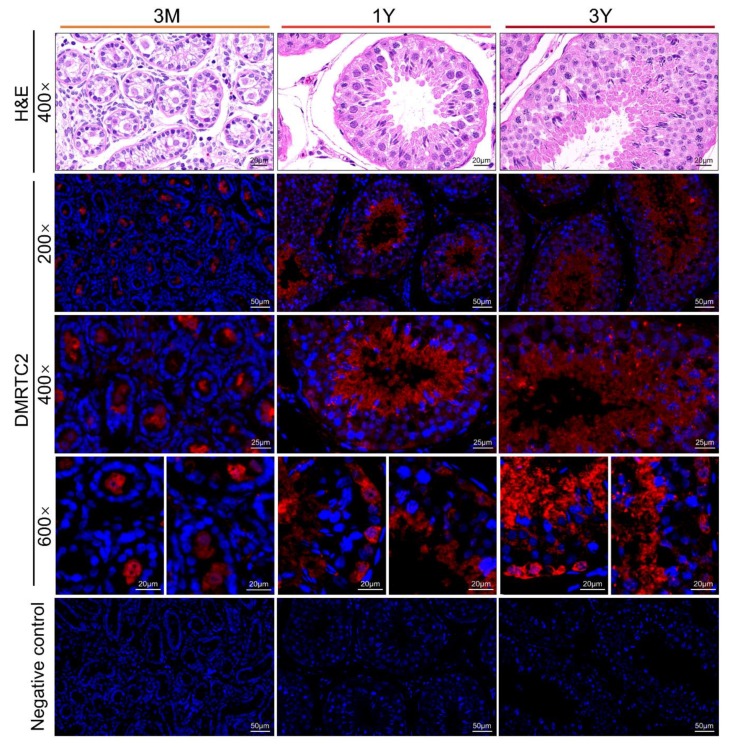
Hematoxylin and eosin (H&E) staining and immunofluorescence staining of DMRTC2 protein within Tibetan sheep testes at different development stages. Nuclei were counterstained with DAPI (blue). 3 M: three months old, 1 Y: one year old, and 3 Y: three years old.

Testicular tissues at all three development stages were constituted of seminiferous tubules and the interstitial tissues between seminiferous tubules (Figure 7). In the 3 M group, the seminiferous tubules were undifferentiated, which were lined with gonocytes and spermatogonia (Figure 7). In 1 Y and 3 Y groups, the seminiferous tubules had obvious lumens and showed all stages of spermatogenic cells, including spermatogonia, primary spermatocytes, secondary spermatocytes, spermatids, and spermatozoa (Figure 7). Compared with the 1 Y group, the 3 Y group had an increased number of layers of spermatogenic cells (Figure 7).

To explore the potential roles of DMRTC2 protein during Tibetan sheep spermatogenesis, the positive DMRTC2 protein signals were detected in developmental testes by using immunofluorescence. Results showed that DMRTC2 protein was located in the seminiferous epithelium throughout developmental stages (Figure 7). For the 3 M group, DMRTC2 protein was mainly observed in gonocytes, while it was mainly observed in spermatids and spermatogonia within the tubule lumens for 1 Y and 3 Y groups, with intense signals in spermatids (Figure 7).

## 3. Discussion

Although the *DMRTC2* gene has been reported to be exclusively expressed in male germ cells and is involved in regulating mammalian spermatogenesis [6,11], its expression and regulation vary depending on species and developmental stages. Additionally, there is still no cure or understanding of molecular characteristics, expression and regulation of *DMRTC2* in Tibetan sheep. In this work, so we firstly cloned the cDNA sequence of Tibetan sheep *DMRTC2* gene. The results revealed that full-length cDNA sequence (1178 bp) consisted of a 1113 bp ORF encoding a protein of 370 deduced amino acids, which is consistent with a previous study in yak and cattle-yak [16]. Additionally, this CDS sequence displayed a high level of homology (no less than 90%) to other published mammalian *DMRTC2* sequences originated from NCBI. These findings suggest *DMRTC2* is highly conserved. To our knowledge, this is the first report on the sequence characteristics of *DMRTC2* in sheep. Compared with the predicted nucleotide sequence available on NCBI database, a three-base (GAG) at position 457 correspond to a single amino acid insertion, and a synonymous C→T mutation at position 831 were found in the cloned Tibetan sheep *DMRTC2* CDS. This discrepancy may be caused by the inaccuracy of predicted sequence obtained from NCBI. Additionally, breed differences may contribute to the discrepancy as well. Nevertheless, the specific reasons for this remain to be further investigated in the future.

The proteins encoded by *DMRT* gene family members, including *DMRTC2*, share a distinctive zinc-finger DNA binding motif called the DM domain [17], which has been shown to be indispensable for mammalian sexual reproduction, such as sexual differentiation [18,19], gametogenesis [20] and gonadal development [20]. As in other mammals such as chiru, goat and cattle, in this study, a DM domain with 47 amino acids in Tibetan sheep DMRTC2 protein was also identified, which is in full concordance with those reported previously in water buffalo [21]. Taken together, ovine *DMRTC2* gene can encode a protein, which contains a DM domain that is very well conserved among mammals.

Understanding the spatial–temporal expression patterns of gene is helpful in unscrambling its functions during the development of an organism. Thus, we subsequently performed qPCR to investigate the temporal patterns of *DMRTC2* gene in male Tibetan sheep reproductive organs during sexual development. Results showed that the transcript abundance for *DMRTC2* was found to be high in Tibetan sheep testes while it was barely detectable in epididymides, which is consistent with the results in previously published research document that *DMRTC2* is a testicular-specific gene in mammals [11,22], suggestive of a role in Tibetan sheep testis. In development Tibetan sheep testes, *DMRTC2* transcript was up-regulated with age, this is in good agreement with earlier reports in postnatal mice testes [11]. To examine whether the DMRTC2 protein shares the similar or same expression patterns with that at the transcript level, we performed Western blot assay with a primary antibody against DMRTC2. As it was expected, DMRTC2 protein expressed in Tibetan sheep testes at all stages of development, and its expression patterns were basically consistent with *DMRTC2* transcript, with obvious abundance in post-pubertal testes. Based on these findings, we speculated that DMRTC2 may exert important functions in sheep testis, and the variable temporal expression patterns of DMRTC2 may be related to its functional divergences during testicular development.

Some previous studies have reported that *DMRTC2* gene is associated with male fertility. For instance, in mice, the mutation or deletion of *DMRTC2* result in male infertility, specifically manifesting in the arrest of spermatogenesis and absence of spermatids [7,11,12]. Kim et al. [11] documented that *DMRTC2* plays a vital role in the recruitment of chromatin regulatory complexes to the sex chromosomes and its mutation gives rise to sex chromatin defects. In cattle, Yan et al. [8] reported that *DMRTC2* highly expressed in adult cattle and yak testes, while only weak *DMRTC2* is identified in the testis of cattleyak that is characterized by spermatogenic arrest and male sterility. In order to further explore the potential roles for *DMRTC2* during sheep spermatogenesis and germ cell development, we detected the patterns of cellular distribution of DMRTC2 protein in developmental Tibetan sheep testes. As was suggested by immunofluorescence analyses, the strong positive signal for the DMRTC2 protein was observed to be mainly present in spermatids from post-pubertal (1 Y and 3 Y) sheep testes. The similar finding has also reported in previous studies [11], which documents that the intense DMRTC2 protein is located in spermatids in adult mice testis. These results are indicative of a possible role for the *DMRTC2* gene in further development of post-meiotic germ cells in Tibetan sheep testis. In addition, DMRTC2 protein signal was observed in gonocytes from pre-pubertal (3 M) sheep testes as well as spermatogonia from post-pubertal sheep testes. Similarly, in human testis, Jan et al. [23] also report that *DMRTC2* is already expressed in spermatogonia, although it is a meiosis-related gene. Moreover, *DMRTC2* is reported to play a regulatory role in the conversion of human gonocyte to type A spermatogonia [14]. As we know from the above, *DMRTC2* might also be shown to be implicated in the differentiation of gonocytes into spermatogonia during sheep spermatogenesis. However, the specific mechanism still requires further exploration in cell and animal experiments.

## 4. Materials and Methods

### 4.1. Animals and Sample Collection

A total of 24 healthy male Tibetan sheep from the same father, at three reproductive stages—pre-puberty (three months old, 3 M; *n* = 8), puberty (one year old, 1 Y; *n* = 8), and post-puberty (three years old, 3 Y; *n* = 8)—were obtained from Xike Tibetan Sheep Breeding Base (Xiahe, China). After sheep were slaughtered, the right testes, caput, corpus, and cauda epididymides were dissected from each ram and then washed with PBS to remove blood. Collected tissues were fixed in 4% paraformaldehyde (Solarbio, Beijing, China) for 48 h used for the embedding procedures or directly frozen in liquid nitrogen and kept at −80 °C until RNA and protein extraction. All animal experiments were approved by the Ethical Committee of Experimental Animal Center of Gansu Agricultural University in compliance with the National Guidelines for Experimental Animal Welfare (Approval No. 2006-398; 306-05-2006-025, 30 Sep 2006).

### 4.2. RNA Isolation and cDNA Synthesis

Total RNA was isolated using Trizol reagent (TransGen, Beijing, China) from the following samples: testis, caput, corpus, and cauda epididymis. The quantity and quality of the total RNA was assessed by NanoDrop ND-2000 spectrophotometer (Thermo Scientific, Niederelbert, Germany) and Agilent 2100 Bioanalyzer (Agilent Technologies, Santa Clara, CA, USA), respectively. One microgram of each RNA sample was reverse-transcribed into cDNA using a TransScript II All-in-One First-Strand cDNA Synthesis SuperMix (TransGen Biotech, Beijing, China), according to the instructions of the manufacturer.

### 4.3. Full-length cDNA Cloning of DMRTC2 Gene

Primer pairs were designed with Primer Premier 6.0 software (Premier Biosoft International, Palo Alto, CA, USA) according to ovine mRNA sequences acquired from GenBank with Accession no. XM_027978391.1. Their specificity was evaluated using the basic local alignment search tool (BLAST) online program (NCBI, U.S. Library of Medicine, Bethesda, MD, USA; http://blast.ncbi.nlm.nih.gov/Blast.cgi). Primers used in this study were synthesized commercially by Tsingke Biotechnology Co. Ltd. (Xi’an, China). The details of the primers are summarized in Table 1. The cDNA derived from testis samples was used as the template to clone the CDS sequences. The PCR reactions were carried out in a final volume of 50 mL consisting of 25 μL of 2× TransStart FastPfu Fly PCR SuperMix (TransGen, Beijing, China), 1 μL of cDNA, 2 μL 10 μM each of forward and reverse primers, 10 μL of PCR Stimulant (TransGen, Beijing, China), and 10 μL of ddH_2_O. The reaction was 95 °C for 5 min, then 30 cycles of 95 °C for 30 s, 55 °C for 30 s, and 72 °C for 1 min. Amplified cDNA products were separated, purified, and then inserted into the pEASY-Blunt vector with a pEASY-Blunt Cloning kit (TransGen, Beijing, China). Subsequently, the ligation product of the amplified fragment with the linear vector pEASY- Blunt was transformed into Trans1-T1 competent cells (TransGen, Beijing, China) following the manufacturer’s instructions. The positive clones were selected on LB-ampicillin plates, and identified by PCR using a 2 × EasyTaq PCR SuperMix (TransGen, Beijing, China). Four independent positive clones from each individual, total of 96 clones, were arbitrarily selected and sequenced by the Tsingke Biotechnology Co. Ltd. (Xi’an, China). The cloned cDNA sequence of DMRTC2 gene was submitted to GenBank of NCBI (http://www.ncbi.nlm.nih.gov).

### 4.4. Bioinformatics Analysis

The homology search of DMRTC2 nucleotide sequence was performed with the BLAST algorithm (NCBI, U.S. Library of Medicine, Bethesda, MD, USA; http://blast.ncbi.nlm.nih.gov/Blast.cgi). Multiple alignments of the DMRTC2 CDS sequence from different species were performed using DNAMAN 8.0 software (Lynnon Biosoft, San Ramon, CA, USA). The entire ORF sequence of DMRTC2 gene was searched with the online ExPASy Translate tool (Swiss Institute of Bioinformatics, Basel, Switzerland; https://web.expasy.org/translate/). Physicochemical properties of the deduced DMRTC2 protein were determined using the ProtParam Server (Swiss Institute of Bioinformatics, Basel, Switzerland; https://web.expasy.org/protparam/). Transmembrane domains were predicted with TMHMM 2.0 online program (Center for Biological Sequence Analysis, Technical University of Denmark, Lyngby, Denmark; http://www.cbs.dtu.dk/services/TMHMM-2.0). The phosphorylated sites were predicted by NetPhos 3.1 Server (Center for Biological Sequence Analysis, Technical University of Denmark, Lyngby, Denmark; http://www.cbs.dtu.dk/services/NetPhos-3.1). The signal peptide was predicted by the SignalP 4.0 Server (Center for Biological Sequence Analysis, Technical University of Denmark, Lyngby, Denmark; http://www.cbs.dtu.dk/services/SignalP-4.0/) [24]. The secondary and tertiary structures of DMRTC2 protein were predicted by using online tools SOPMA (Institute of Biology and Chemistry of Proteins, Lyon, France; https://npsa-prabi.ibcp.fr/cgi-bin/npsa_automat.pl?page=npsa_sopma.html) and Phyre 2.0 (Structural Bioinformatics Group, Imperial College London, London, UK; http://www.sbg.bio.ic.ac.uk/phyre2/html/page.cgi?id=index), respectively. The phylogenetic tree was constructed using the neighbor-joining (NJ) method from MEGA 7.0 software (Institute for Genomics and Evolutionary Medicine, Temple University, Philadelphia, PA, USA) [25], and the reliability of each branch was tested maximum-likelihood method (1000 bootstrap replications). The domain of DMRTC2 protein was predicted by the Conserved Domain Database (http://www.ncbi.nlm.nih.gov/Structure/cdd/wrpsb.cgi) [26] at the NCBI server, and the sequence logo of DM domain was visualized using the WebLogo 3.7 online tool (University of California, Berkeley, CA, USA; http://weblogo.threeplusone.com/).

### 4.5. Quantitative Real-time PCR (qPCR)

The qPCR experiment was conducted on a LightCycler 96 Real-Time System (Roche, Basel, Switzerland) using the following procedures: 94 °C for 30 s; 40 cycles of 94 °C for 5 s; and 60 °C for 30 s. Each PCR reaction system (20 μL) contained: 10 μL of 2× TransStart Tip Green qPCR SuperMix (TransGen, Beijing, China); 0.8 μL of cDNA, 0.4 μL of each primer (10 μM; Table 1), and 8.4 μL of ddH_2_O. All reactions were run in triplicate within each of eight biological replicates. The mRNA expression of DMRTC2 was calculated relative to that of β-actin used for a housekeeping gene by the 2^−ΔΔCt^ method [27].

### 4.6. Western Blot

Western blot assay was performed for detecting the expression of DMRTC2 protein. Samples of sheep testicular tissues were homogenized lysed with a radio immunoprecipitation assay (RIPA) protein extraction kit (Solarbio, Beijing, China) containing phenylmethanesulfonyl fluoride (PMSF; Solarbio, Beijing, China). The extracted protein was quantified using a commercial bicinchoninic acid (BCA) kit (Beyotime, Shanghai, China). The denatured proteins (15 µg) were separated by 12% (w/v) sodium dodecyl sulfate polyacrylamide gel electrophoresis (SDS-PAGE) gels, electrotransferred onto polyvinylidene fluoride (PVDF) membrane (Immobilon-P Transfer Membrane, Merck Millipore, Tullagreen, Ireland), and then blocked in 5% (g/mL) non-fat milk made in Tris-buffered saline plus tween (TBST) before being incubated with either rabbit anti-DMRTD2 polyclonal antibody (1:500 dilution; Bioss, Beijing, China) or anti-beta-actin polyclonal antibody (1:1500 dilution; Bioss, Beijing, China) overnight at 4 °C. The membranes were incubated with a goat anti-rabbit IgG conjugated with horseradish peroxidase (HRP; 1:1500 dilution; Bioss, Beijing, China) for 1.5 h at room temperature. Experiments were performed in eight biological replicates, and each replicate included two technical replicates. The signals of bands were visualized using an enhanced chemiluminescence (ECL) kit (NCM Biotech, Suzhou, China). The band intensities were assessed by an AlphaEaseFC software (Protein Simple, Santa Clara, CA, USA). Relative expression of DMRTC2 protein was calculated by the average intensity of DMRTC2 bands relative to β-actin bands.

### 4.7. Histologic and Immunofluorescence Analysis

For histologic analysis, the paraffin sections of testes from all sheep at three development stages were prepared and stained with hematoxylin and eosin (H&E) using conventional methods as previously described [28], with some minor modifications. The paraffin sections from testicular tissues were processed to examine the cellular localization of DMRTC2 protein by using immunofluorescence as previously described [29]. In brief, sections were subjected to conventional dewaxing and gradient ethanol dehydration, followed by antigen repair. After being blocked in 5% bovine serum albumin (BSA; Solarbio, Beijing, China), sections were incubated with polyclonal rabbit primary antibody against DMRTC2 (1:200 dilution; Bioss, Beijing, China) overnight at 4 °C. Subsequently, sections were incubated with CY3-labeled goat anti-rabbit IgG (1:200; Servicebio, Wuhan, China) for 1 h at room temperature in the dark. Sections were washed thrice with PBS and treated with 4′,6-diamidino-2-phenylindole (DAPI; Servicebio, Wuhan, China) for 10 min in the dark. After sealing, the slides were placed under a fluorescence microscope (Nikon, Eclipse C1, Tokyo, Japan) for observation. For negative control slides, primary antibody was only replaced with 5% BSA, and the other conditions and steps were the same. Experiments were done on eight biological replicates with two technical replicates each. Digital images were captured using a CaseViewer software (3DHISTECH, Budapest, Hungary).

### 4.8. Statistical Analysis

All experiments were repeated at least three times. The relative expression levels of DMRTC2 mRNA and protein were statistically analyzed using one-way analysis of variance in SPSS 21.0 (SPSS Inc., Chicago, IL, USA). All data are shown in the form of bar charts as mean ± standard deviation (SD). Differences with *p* < 0.05 and *p* < 0.01 were regarded as statistically significant and very significant, respectively.

## 5. Conclusions

In conclusion, this is the first report regarding molecular cloning and characteristics of the ovine *DMRTC2* CDS region, as well as describing its expression patterns and potential roles during Tibetan sheep spermatogenesis. The full-length CDS of the Tibetan sheep *DMRTC2* gene was 1113-bp-long and encoded 370 amino acid residues containing a conserved DM domain of 47 amino acids. *DMRTC2* specifically expressed in Tibetan sheep testis, with an age-dependent augmented expression pattern. DMRTC2 protein was present in gonocytes in pre-puberty Tibetan sheep testis, and spermatids and spermatogonia in post-puberty Tibetan sheep testis. On the basis of these findings, we concluded that *DMRTC2* might play roles in the proliferation or differentiation of gonocytes and development of spermatids.

## Figures and Tables

**Table 1:** List of the primers used in this study.

Gene	Accession *no*.	Primer Sequence (5′–3′)	Size (bp)	Purpose
*DMRTC2*	XM_027978391.1	F: ATGGACCCCAATGAAATGCC	1178	cDNA cloning
		R: CTAGCTTAGGAGGGAAATGG		
*DMRTC2*	XM_027978391.1	F: CTGAGGCTCTTGTGGGACTG	96	qPCR
		R:CACAAGGATGGAGCAGGGAG		
*β-actin*	NM_001009784.2	F: CTTCCAGCCTTCCTTCCTGG	180	qPCR
		R: GCCAGGGCAGTGATCTCTTT		

F, forward primer; R, reverse primer.

## Supplementary Materials

Supplementary materials can be found at https://www.mdpi.com/1422-0067/21/7/2448/s1.

## Abbreviations

DMRTC2	Double sex and mab-3-related transcription factors like family C2
ORF	Open reading frame
qPCR	Quantitative real-time PCR
RIPA	Radio immunoprecipitation assay
BCA	Bicinchoninic acid
SDS-PAGE	Sodium dodecyl sulfate polyacrylamide gel electrophoresis
