# Supplementary material for: Cloning, Molecular Characterization and Expression Patterns of DMRTC2 Implicated in Germ Cell Development of Male Tibetan Sheep
Source: Int J Mol Sci. 2020 Apr 1;21(7):2448. doi: 10.3390/ijms21072448 (PMC7177445; doi:10.3390/ijms21072448)
Supplement: Supplementary file 1 [file ijms-21-02448-s001.pdf]

Supplementary file

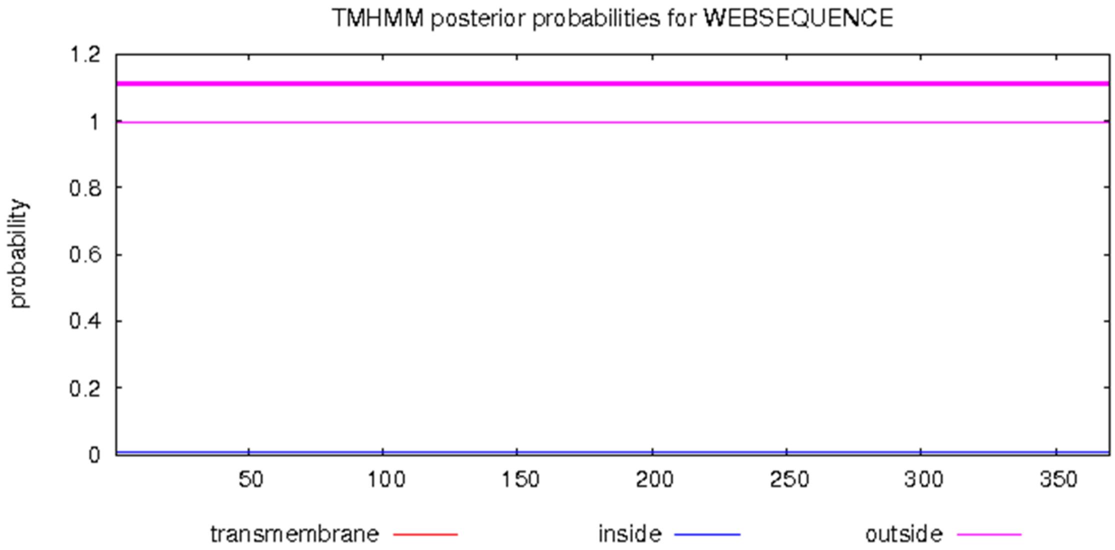

Figure S1. Prediction of transmembrane region of Tibetan sheep DMRTC2 protein.

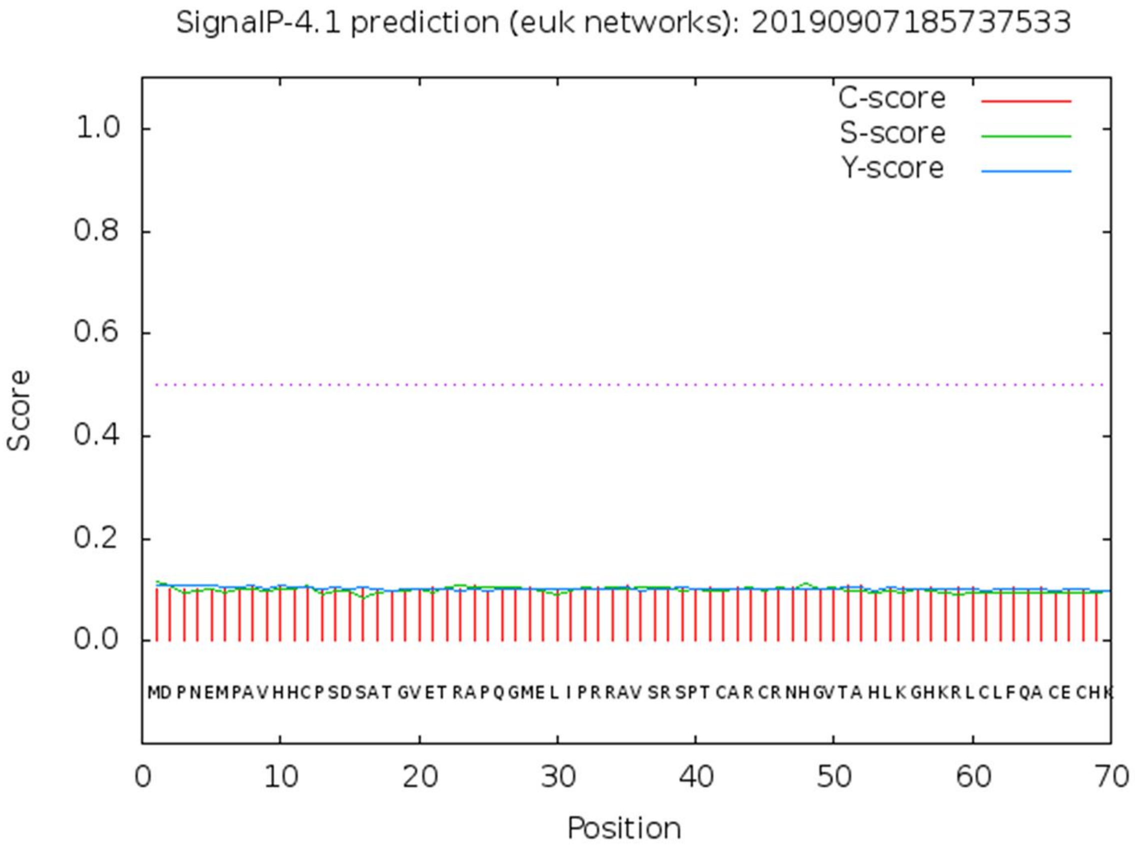

Figure S2. Prediction of signal peptide of Tibetan sheep DMRTC2 protein.

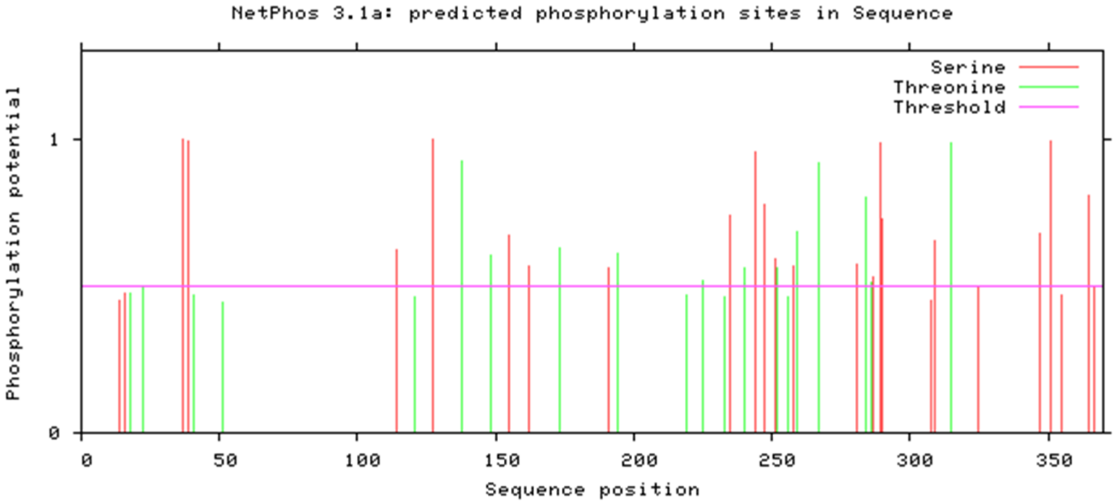

Figure S3. Prediction of phosphorylation sites of Tibetan sheep DMRTC2 protein.

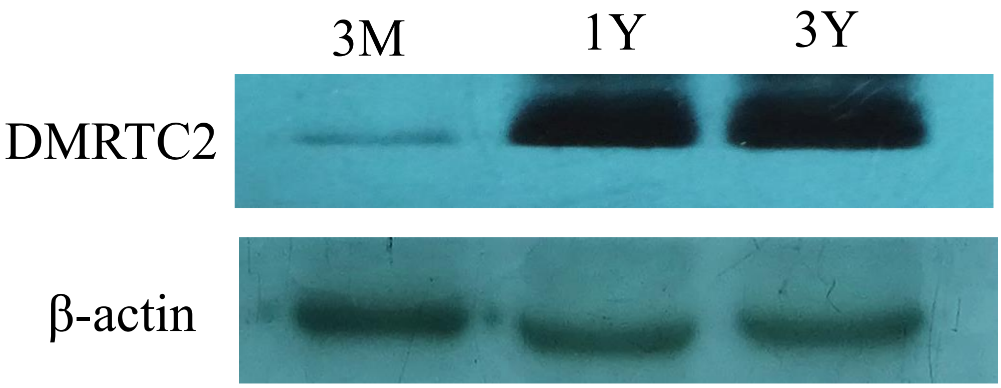

Figure S4. The full blot images for Tibetan sheep DMRTC2 and β-actin.
